# On Best Erasure Wiretap Codes: Equivocation Matrices and Design Principles

**DOI:** 10.3390/e27121245

**Published:** 2025-12-09

**Authors:** Willie K. Harrison, Truman Welling, Andrew Swain, Morteza Shoushtari

**Affiliations:** Department of Electrical and Computer Engineering, Brigham Young University, Provo, UT 84602, USA; truman.welling@gmail.com (T.W.); swain.andrew.g@gmail.com (A.S.); morteza.shoushtari@byu.edu (M.S.)

**Keywords:** information theoretic security, equivocation matrices, wiretap coset codes, finite blocklength analysis

## Abstract

Physical-layer security can aid in establishing secure telecommunication networks including cellular, Internet of Things, and telemetry networks, among others. Channel sounding techniques and/or telemetry systems for reporting channel conditions, coupled with superior wiretap code design are necessary to implement such secure systems. In this paper, we present recent results in best wiretap coset code design for the binary erasure wiretap channel. We define equivocation matrices, and showcase their properties and utility in constructing good, and even the best, wiretap codes. We outline the notion of equivalence for wiretap coset codes, and use it to reduce the search space in exhaustive searches for best small codes. Through example, we show that the best codes do not exist for some code sizes. We also prove that simplex codes are better than codes repeating one column multiple times in their generator matrix.

## 1. Introduction

Wireless telecommunications are susceptible to eavesdropping due to their broadcast nature [1,2]. For cellular communications, it is expected that 6G networks will mitigate some of the threat of eavesdropping using beamforming/directional communications [3], but this will not solve the problem entirely. Additionally, networks of all kinds, not just cellular, exhibit this vulnerability. The IoT in general promises to place a heavy emphasis on small packet communications with low latency, low power, and high reliability requirements [4]. Unfortunately, the well-tested public-key/private-key cryptographic approaches [5] do not scale well to networks with these characteristics [6,7]. There is an urgent need for robust and adaptable security solutions for these new networks if they are to be trusted with future sensitive communications [6,7,8,9].

One option for addressing these security needs is physical-layer security [10], wherein the characteristics of the wireless channel are exploited for security gains. A host of recent papers [11,12,13,14,15] outline the multi-pronged approach needed to achieve physical-layer security. On one hand, system designers must test the environment using channel sounding techniques, which could include real-time telemetry systems that communicate channel conditions from various nodes in the network. On the other hand, good wiretap codes are needed to exploit favorable channel conditions for physical-layer security. This paper addresses the second aspect of implementing systems capable of achieving physical-layer security by addressing wiretap code design at finite blocklength.

**Figure 1:**
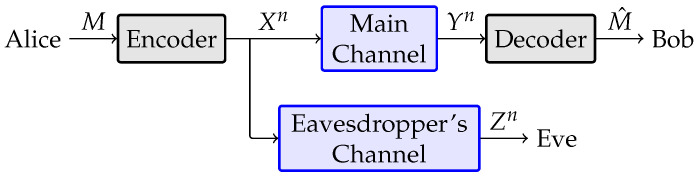
Wiretap channel model.

Achieving information theoretic security over wiretap channels has been a topic of interest for several decades now [16,17]. Many works have focused on the fundamental limits of physical-layer security over various channels [10,18], but several others have tried to find explicit coding constructions for achieving those limits (see [19,20] and references). To date, the majority of coding results require codes to be analyzed in the asymptotic blocklength regime, mainly since the common information theoretic security metrics are analyzed in the limit as blocklength tends to infinity. Consider the wiretap channel model in Figure 1, where a user named Alice attempts to communicate a message *M* reliably over a *main channel* of communications to a user named Bob without leaking information to an eavesdropper named Eve over the *eavesdropper’s channel*. Alice encodes *M* using code C to produce a length-*n* codeword $X^{n}$, which is broadcast over both the main and the eavesdropper’s channels. Bob and Eve observe $Y^{n}$ and $Z^{n}$ through their respective channels, and Bob’s estimate of the message is denoted $\hat{M}$ (note that we will use capital letters to denote random variables/vectors, with matching lowercase letters signifying realizations of those variables and matching calligraphic letters signifying the alphabets over which those variables are defined). Coding over the wiretap channel is typically carried out with two goals in mind. A reliability constraint on the problem requires $\lim_{n\to \infty }\mathrm{Pr}\left(\hat{M}\neq M\right)=0$, and a security constraint based on information theory is imposed on the problem as well. The following security constraints are perhaps the most popular [10,21]:

- $\lim_{n\to \infty }\frac{1}{n}I\left(M;Z^{n}\right)=0$, where $M∼U[0,2^{k}-1]$, (weak secrecy);
- $\lim_{n\to \infty }I\left(M;Z^{n}\right)=0$, where $M∼U[0,2^{k}-1]$, (strong secrecy);
- $\lim_{n\to \infty }\max_{p_{M}\left(m\right)}I\left(M;Z^{n}\right)=0$, (semantic secrecy).

In these definitions, I(·;·) is the usual mutual information function [22], $M∼U[0,2^{k}-1]$ signifies that the message is distributed as discrete uniform over the integers $〚0,2^{k}-1〛=\left\{0,1,\ldots ,2^{k}-1\right\}$, and $p_{M}\left(m\right)$ is the probability mass function of *M*. In all three constraints, the range of *M* is taken to be $M=〚0,2^{k}-1〛$.

If finite blocklength wiretap codes are to be optimized, or even compared with each other, these asymptotic metrics are often insufficient. In this paper we consider only finite blocklength wiretap codes based on the cosets of a linear block code [17,23,24], and fix *n* to be relatively small as compared to blocklengths commonly encountered in error-control coding [25,26,27]. The design constraints for our fixed blocklength analysis are:

1. $\mathrm{Pr}\left(\hat{M}\neq M\right)<\delta_{m}$, (reliability constraint);
2. $I\left(M;Z^{n}\right)<\delta_{e}$, where $M∼U[0,2^{k}-1]$, (security constraint),

and $\delta_{m}$ and $\delta_{e}$ are assumed to be small positive real numbers. Note that

(1) $$I\left(M;Z^{n}\right)=H\left(M\right)-H\left(M|Z^{n}\right),$$

where H(·) is the average entropy function [22], and

(2) $$E=H\left(M|Z^{n}\right)=\sum_{z^{n}\in Z^{n}}p\left(z^{n}\right)H\left(M|Z^{n}=z^{n}\right)$$

is called the *equivocation*. The goal of this current line of inquiry is to discover the best binary codes of a particular size, wherein we define a *best code* as follows.

**Definition** **1.** *For fixed parameters n and k, a code that minimizes $I(M;Z^{n})$ (or, equivalently, maximizes $H(M|Z^{n})$)* for all possible eavesdropper’s channel states *over the choice of binary wiretap codes with parameters n and k is termed* best *or* best for its size. *If no code with parameters n and k maximizes the equivocation for all possible eavesdropper’s channel states, then we say that a best code* does not exist *for code parameters n and k. We restrict our analysis to wiretap codes that are built using the well-known coset coding structure.*

Knowledge of the best codes, where they exist, will allow engineers to maximize the secrecy in a communications network without worrying about achieving asymptotic security constraints. Furthermore, asymptotic security guarantees can only be made with knowledge of the eavesdropper’s channel state information (CSI) [10,19,20], which is often not readily available during code design anyway. Perhaps a better approach is to simply set the code parameters, and then maximize the achievable secrecy with the choice of a best code [12] (note here that our techniques will also easily allow a worst-case leakage analysis to be completed, thereby also covering the wiretap-II case [23]).

A few works [28,29,30,31] have attempted to discover the best wiretap codes, but the results are somewhat inconclusive as the authors assume that large best codes can be grown from small best codes, which is an unproven assumption. A local search algorithm using a smaller code as a seed then provides the results for the larger code parameters. Our approach to best coset coding, however, is to provably identify codes with best performance and their properties [32,33,34,35,36]. Such research requires the analysis of algebraic properties of encoders along the lines of the work in [24,37,38]. Some other works have also sought to quantify the information theoretic security as a function of blocklength [39,40], but the results are only bounds that are quite pessimistic for small to medium blocklength codes. Our approach differs from these works in that we seek to quantify the equivocation exactly as we identify the best codes.

This paper analyzes coset codes over the simplest wiretap channel model, where the main channel is noiseless and the eavesdropper’s channel is a binary erasure channel (BEC); this model is called the binary erasure wiretap channel (BEWC). However, just as error-control coding results for the BEC have led to more powerful results over real-world channels (e.g., Gaussian channels) [27], it is expected that good (and even best) codes found while studying the BEWC will lead to optimal code structures for more interesting channels [35,41]. Note that focusing on this model allows us to effectively ignore the reliability constraint since $\mathrm{Pr}(\hat{M}\neq M)=0$ for all valid codes; thus, it becomes possible to rank codes only according to how well they keep information secret from Eve.

The main contributions of this paper build on those previously presented in [32,33,34,35,36], and are as follows:

- A complete presentation of the equivocation matrix and its properties as a tool for finite blocklength analysis and code design over the BEWC (the tool has been used in [33,34] but will benefit from this more complete discussion);
- An explanation of search space reduction techniques that allow for the discovery of best small blocklength codes (the results from these techniques were shown in [32,33,35], but the techniques have not yet been explained in the literature);
- A discussion of the results regarding the existence of best coset codes for information theoretic security (it has been hypothesized that a best code exists for all valid (n,k) pairs in [33], but this is proved otherwise herein by way of counterexample);
- A high-level algorithm for the design of good wiretap coset codes for the BEWC of any size that follows an *outside-in* approach to code design that is backed by equivocation matrix properties for best codes;
- Results related to simplex and Hamming codes: notably that the simplex code is better for *all* eavesdropper channel states than any code of its size with exactly one column of the generator matrix repeated any number of times.

The rest of this paper is organized as follows. Section 2 presents the necessary background information required for the remainder of this paper, including an explanation of encoding and decoding using wiretap codes for the BEWC based on the cosets of a linear block code, and a sufficient condition for classifying a binary coset code as *best* that is easier to work with than Definition 1. Then, Section 3, Section 4, Section 5, Section 6 and Section 7 give the main results of this paper. Equivocation matrices and their properties are defined and discussed in Section 3. Search space reduction techniques for finding small best codes are presented in Section 4. Section 5 highlights our current knowledge regarding the existence of best codes. Section 6 shows how to build good codes of any size and links the method to properties of best codes’ equivocation matrices. Finally, the new result regarding the optimality of simplex and Hamming codes is presented in Section 7. This paper is concluded in Section 8.

## 2. Background

### 2.1. Coset Coding for the Wiretap Channel

The binning technique known as coset coding used in many prior works [17,23,42] is the coding mechanism analyzed in this paper. Let C denote an (n,n−k) linear block code, and $C_{0},C_{1},\ldots ,C_{2^{k}-1}$ signify the cosets of C, where $C_{0}=C$. The generator and parity-check matrices for C are denoted *G* and *H*, respectively. The encoder function encodes the message *m* by choosing a codeword from $C_{m}$ uniformly at random. This operation can be conducted using an auxiliary message $M^{\prime }$.

The auxiliary message is an (n−k)-bit row vector that carries no real information, and is chosen uniformly at random from the elements in the field of length-(n−k) binary vectors $F_{2}^{\left(n-k\right)}$. Let *m* be written in the form of a *k*-bit binary row vector. Also let

(3) $$G^{*}=\left[\begin{array}{c} G\\ G^{\prime } \end{array}\right],$$

where the rows of $G^{\prime }$ are chosen to give $G^{*}$ full rank, and must therefore be linearly independent and chosen from outside C. The codeword is calculated as

(4) $$x^{n}=\left[\begin{array}{cc} m^{\prime } & m \end{array}\right]\left[\begin{array}{c} G\\ G^{\prime } \end{array}\right]=m^{\prime }G+mG^{\prime },$$

and the mathematical encoding operation is carried out in $F_{2}$. Clearly, $m^{\prime }G$ chooses a codeword from C, and $mG^{\prime }$ chooses an offset that maps the codeword into a specific coset of C. Thus, *m* chooses the coset, and $m^{\prime }$ chooses the codeword from the coset uniformly at random.

For example, let

(5) $$G^{*}=\left[\begin{array}{c} G\\ G^{\prime } \end{array}\right]=\left[\begin{array}{cccc} 1 & 1 & 0 & 1\\ 0 & 0 & 1 & 1\\ 1 & 1 & 0 & 0\\ 1 & 0 & 1 & 0 \end{array}\right].$$

This encoder defines the code in Table 1.

Since we are operating over the BEWC, the main channel is noiseless and Bob needs only to map the codeword back to the proper message to do the decoding. The decoder function first calculates a syndrome

(6) $$s=y^{n}H^{T}=x^{n}H^{T}=m^{\prime }GH^{T}+mG^{\prime }H^{T}=mG^{\prime }H^{T}.$$

Notice that $G^{\prime }H^{T}$ forms a bijective mapping between *s* and *m*. If $G^{\prime }$ and *H* are chosen so that $G^{\prime }H^{T}=I_{k}$, the (k×k) identity, then s=m. Otherwise, the mapping will need to be inverted to complete the decoder [32]. For our example code, let

(7) $$H=\left[\begin{array}{cccc} 1 & 0 & 1 & 1\\ 1 & 1 & 0 & 0 \end{array}\right].$$

It can be verified that $G^{\prime }H^{T}=I_{2}$; so, s=m in this case.

### 2.2. Equivocation for Coset Codes

Let us write the generator matrix for C in terms of its columns as

(8) $$G=\left[\begin{array}{cccc} g_{1} & g_{2} & \ldots & g_{n} \end{array}\right].$$

Also, for a set of integers $J=\{j_{1},j_{2},\ldots ,j_{\left|J\right|}\}$ such that $1\leq j_{i}\leq n$ for i∈〚1,|J|〛, let

(9) $$G_{J}=\left[\begin{array}{cccc} g_{j_{1}} & g_{j_{2}} & \ldots & g_{j_{\left|J\right|}} \end{array}\right];$$

i.e., $G_{J}$ is the submatrix of *G* comprising only the columns with indices in *J*. Note that the notation |J| indicates the cardinality of the set *J*. Since $Z^{n}$ is Eve’s observation of the transmitted codeword through a BEC, then $Z^{n}={\left\{0,1,?\right\}}^{n}$, where `?’ indicates an erased bit. Let

(10) $$r\left(z^{n}\right)=\left\{i|z_{i}\neq \;?\right\};$$

thus, $r(z^{n})$ is the set of indices for all revealed bits to Eve in $z^{n}$. It was shown in [32,35] that

(11) $$\begin{array}{cc} H(M|Z^{n}=z^{n}) & =H\left(M\right)-|r\left(z^{n}\right)|+\mathrm{rank}\left(G_{r(z^{n})}\right)\\ \; & =k-|r\left(z^{n}\right)|+\mathrm{rank}\left(G_{r(z^{n})}\right). \end{array}$$

This quantity is the *exact equivocation* given a particular observation $z^{n}$, and it can be used to calculate the average equivocation $H(M|Z^{n})$ in (2) by summing over all possible $z^{n}\in Z^{n}$.

By way of example, let us consider $z^{n}=\left[?\;1\;1\;?\right]$ with the code defined by (5). Then, $r\left(z^{n}\right)=\left\{2,3\right\}$, and $\mathrm{rank}(G_{r(z^{n})})=2$ giving $H(M|Z^{n}=z^{n})=2$ bits from (11). Notice that Table 1 verifies the equivocation at 2 bits since all four cosets have an entry consistent with $z^{n}$, and all four messages are a priori equally likely. However, it is also straightforward to see that if $z^{n}$ were such that $r\left(z^{n}\right)=\left\{1,2\right\}$, then $\mathrm{rank}(G_{r(z^{n})})=1$ and we would expect to leak one bit of information in this case. Again, the code in Table 1 verifies this claim, as any observation that reveals the first two bits will only have two consistent cosets, and thereby one bit of equivocation. It is of note that the particular values of the revealed bits make no difference in the exact equivocation calculation, but rather only the revealed-bit pattern $r(z^{n})$.

### 2.3. A Sufficient Condition for the Existence of Best Wiretap Coset Codes for All ϵ

**Figure 2:**
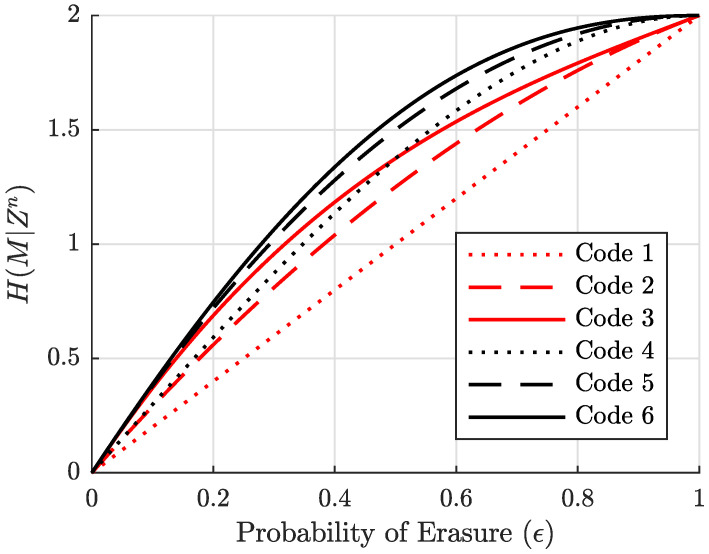
Equivocation of all n=4, k=2 secrecy codes as a function of Eve’s channel’s erasure probability ϵ.

Figure 2 gives equivocation curves for all possible coset codes with n=4 and k=2 as a function of the eavesdropper’s erasure probability ϵ. Note there are only six codes analyzed, but *G* is (n−k)×n (2×4 in this case), and therefore comprises eight symbols. Certainly, we can produce more than six valid generators with eight binary symbols, but there are in fact exactly six codes up to isomorphisms in the code structure. This will be explained further in Section 4. It can be observed in Figure 2 that the equivocation curves of Codes 3 and 4 cross at ϵ=0.5, indicating that codes cannot simply be ordered from best to worst without an operating point for the eavesdropper’s channel, say $\epsilon_{0}$. However, Code 6 gives maximum equivocation over all codes for all ϵ, and hence can be labeled as *best*. These interesting features indicate that if we wish to find best codes, then we must first guarantee the existence of a code that is *best* for all possible eavesdropper’s CSI. As will be shown in Section 5, the existence of a best code for any specific choices of *n* and *k* is not easily deducible. As a note, the equivocation curve for Code 6 is that of the example code given in (5) and Table 1.

Suppose that an (n,n−k) linear block code C with generator *G* is used for coset coding as described in Section 2.1. Let $R_{\mu }$ be the set of all possible revealed-bit patterns *r*, each of which is a subset of 〚1,n〛, such that |r|=μ. Then, using (2) and (11),

(12) $$\begin{array}{cc} H(M|Z^{n}) & =\sum_{z^{n}\in Z^{n}}p\left(z^{n}\right)H\left(M|Z^{n}=z^{n}\right) \end{array}$$

(13) $$\begin{array}{cc} \; & =\sum_{z^{n}\in Z^{n}}p\left(z^{n}\right)\left[H\left(M\right)-|r\left(z^{n}\right)|+\mathrm{rank}\left(G_{r(z^{n})}\right)\right] \end{array}$$

(14) $$\begin{array}{cc} \; & =\sum_{\mu =0}^{n}\sum_{r\in R_{\mu }}{\left(1-\epsilon \right)}^{\mu }\epsilon^{n-\mu }\left[k-\mu +\mathrm{rank}(G_{r})\right]. \end{array}$$

Note that a sufficient condition for a code to maximize (14) for all ϵ, and for fixed *n* and *k*, is for the code to maximize

(15) $$\sum_{r\in R_{\mu }}\mathrm{rank}\left(G_{r}\right)$$

for all μ∈〚0,μ〛 [33]. If such a code exists for specific *n* and *k*, then each iteration of the outer sum of (14) will be as large as possible, thus maximizing the entire equivocation. Such a code is clearly *best* for all possible ϵ.

## 3. Equivocation Matrices and Their Properties

It is now necessary to define the equivocation matrix to continue our study of best codes. Equivocation matrices were first presented in [33] and the basic presentation of the definitions is similar here. In comparing codes of a particular size, it is useful to know the number of revealed-bit patterns with μ revealed bits that maintain *e* bits of equivocation. There are nμ ways to reveal μ of *n* bits through an erasure channel, and all of these patterns must be accounted for when calculating the equivocation $H(M|Z^{n})$.

### 3.1. Basic Definitions

Let us consider the expression for the exact equivocation in (11). Note that all the elements of the expression (*k*, $\left|r(z^{n})\right|$, and $\mathrm{rank}(G_{r(z^{n})})$) are each confined to the set of non-negative integers. Since basic information theory [22] tells us that $H(M|Z^{n}=z^{n})$ must be between zero bits and H(M)=k bits, then the integer elements of (11) further tell us that $H(M|Z^{n}=z^{n})\in 〚0,k〛$. Therefore, since there are no revealed-bit patterns that can leak partial bits, we can count the number of revealed-bit patterns of size μ that maintain equivocation equal to *e* bits and collect this information in a matrix.

**Definition** **2.** *Let the (k+1)×(n+1)* equivocation matrix *A for the (n,k) coset code defined by the (n,n−k) linear block code C denote the number of revealed-bit patterns of size μ that maintain e bits of equivocation in the matrix element $a_{e,\mu }$ for μ∈〚0,n〛 and e∈〚0,k〛. Let $a_{0,0}$ be the bottom left entry of the matrix, and indexing proceed from that point.*

**Figure 3:**
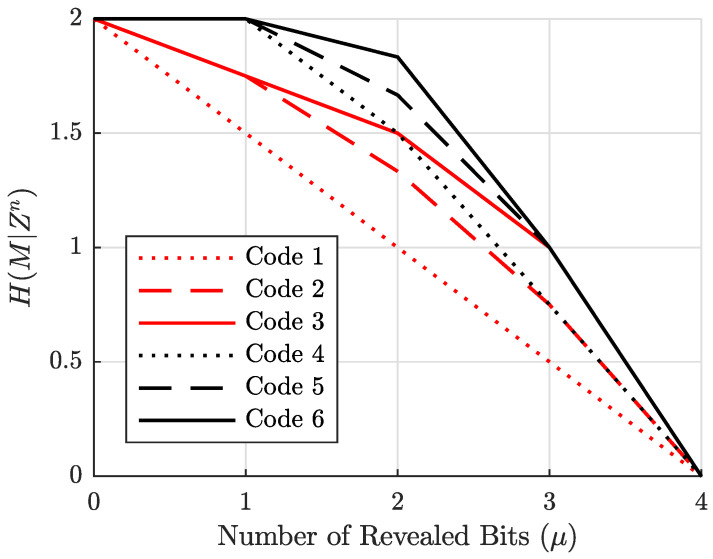
Equivocation of all n=4, k=2 coset codes as a function of the number of revealed bits to Eve μ.

Perhaps somewhat unconventionally, we start indexing the rows of the equivocation matrix from the bottom of the matrix. This is carried out to match plots of *e* vs μ in structure. By way of example, the equivocation matrix for the code defined by (5) and given in Table 1 is

(16) $$A=\left[\begin{array}{ccccc} 1 & 4 & 5 & 0 & 0\\ 0 & 0 & 1 & 4 & 0\\ 0 & 0 & 0 & 0 & 1 \end{array}\right],$$

and the equivocation curves plotting $H(M|Z^{n})$ as a function of the number of revealed bits μ for all coset codes with n=4 and k=2 are given in Figure 3, with our example code being Code 6 as before.

Consider the third column (corresponding to μ=2) of the equivocation matrix. There are 42=6 ways to reveal two of four bits over a BEC; hence, the column sum is six. We see here that five of the six patterns leak no information about the message (equivocation is two bits), but the last pattern leaks one bit about *M*. This pattern was already mentioned in Section 2.2 as $r\left(z^{n}\right)=\left\{1,2\right\}$. The average equivocation for Code 6 plotted in Figure 3 shows that $H(M|Z^{n})=11/6\approx 1.8333$ bits at μ=2. For all other μ values, $H(M|Z^{n})$ is an integer since all revealed-bit patterns of those sizes leak the same number of full bits.

### 3.2. Properties of the Equivocation Matrix

In our discussion of the properties of equivocation matrices, we first mention a finding from [33]. Let us define $A^{⊥}$ as the (n−k+1)×(n+1) equivocation matrix for the (n,n−k) coset code built from the (n,k) dual code $C^{⊥}$, and let the elements of $A^{⊥}$ be $a_{e,\mu }^{⊥}$, just as for *A*, but now letting e∈〚0,n−k〛, and μ∈〚0,n〛 as before.

**Lemma** **1** (Lemma 1 from [33]). *Consider an (n,n−k) linear block code C and its dual code $C^{⊥}$. The equivocation matrices of the coset codes over the BEWC formed by the two linear block codes are related by $a_{e,\mu }=a_{e+\mu -k,n-\mu }^{⊥}.$*

The proof is given in [33], where it is shown that every pattern with μ revealing bits leading to equivocation *e* for coset coding with C, maps precisely to one unique pattern with n−μ revealed bits leading to equivocation e+μ−k for coset coding with $C^{⊥}$. In [43], we extended the result to show that the second pattern is the set complement of the first pattern, and we demonstrated that

(17) $$H\left(M|Z^{n}=z^{n}\right)=\mathrm{rank}\;H_{〚1,n〛\setminus r(z^{n})}.$$

**Figure 4:**
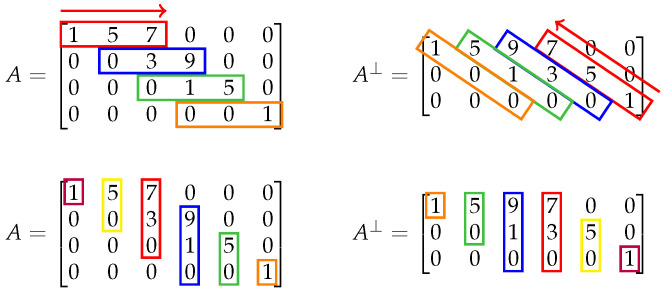
Pictorial representation of the dual relationship between equivocation matrices *A* and $A^{⊥}$. The colored boxes and ordering arrows highlight the locations of identical entries in the matrices for dual codes, which is consistent with Lemma 1.

Figure 4 illustrates the meaning of the lemma when C is the (5,2) code with the generator matrix

(18) $$G=\left[\begin{array}{ccccc} 1 & 0 & 0 & 0 & 1\\ 0 & 1 & 1 & 1 & 1 \end{array}\right].$$

When used for coset coding, this generator matrix produces a coset code with n=5 and k=3. When the dual code is used for coset coding, however, n=5 and k=2. Note that the entries in *A* dictate precisely the entries in $A^{⊥}$, and the placement of the entries in $A^{⊥}$ is as prescribed by Lemma 1.

In [33], it was further noted that all zeros in *A* can either be attributed to the generalized Hamming weights of $C^{⊥}$ (see [24]) or the upper right triangle of zeros, which must be present in every equivocation matrix. Using further arguments from [24], it was shown that all binary maximum distance separable (MDS) codes are *best* codes. In fact, MDS codes are the only binary codes that can guarantee exactly one nonzero entry in *A* per column, and these entries always achieve maximum secrecy for each μ∈〚0,n〛. Thus, they are proved to be best codes because they satisfy the sufficient condition for best by maximizing (15) for all μ∈〚0,μ〛. Due to the structural similarities of *A* and $A^{⊥}$, it was further shown that if C is a best code, then so is $C^{⊥}$. Finally, it was shown in [34] that the equivocation matrix can be completely filled using only knowledge of the full-rank square submatrices in *G*; in other words, knowledge of the revealed-bit patterns counted in $a_{k,n-k}$ is sufficient to completely characterize a code’s equivocation.

**Figure 5:**
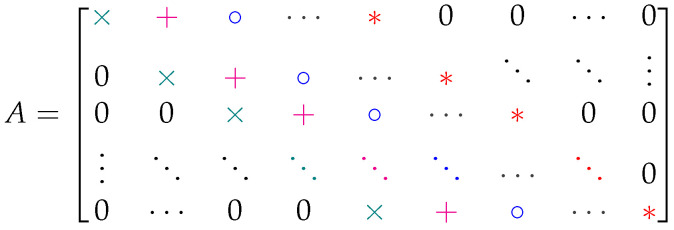
Diagonal properties of the equivocation matrix *A*. All revealed-bit patterns *r* counted in the same diagonal of *A* have the same value for $\mathrm{rank}(G_{r})$; patterns counted in × slots have rank 0, patterns counted in + slots have rank 1, patterns counted in ∘ slots have rank 2,…, patterns counted in * slots have rank *(n − k)*.

There are additional properties of these matrices that make them useful for coset code analysis. For example, it is shown in Figure 5 that revealed-bit patterns *r* counted along the same diagonal of *A* must have the same value for $\mathrm{rank}(G_{r})$. In fact, one can start from the bottom × in Figure 5 and fill out the entire equivocation matrix by looking for sets of columns in *G* with rank zero, then rank one, and so on. Consider, e.g., the largest collection of columns in *G* with rank zero. Each column in this set must be an all-zero column. Thus, all subsets of the set also have rank zero, and can be counted along the rank-zero diagonal for the appropriate size of the pattern. When repeating the exercise for the rank-one diagonal, one can consider large sets of columns in *G* with rank one. These must consist either of identical columns, or zero columns mixed with identical columns. Since the subsets of these sets with only zero columns were already counted in the previous diagonal, they should not be counted again, but all other subsets of these larger sets will have rank one. This procedure can be repeated until the remaining revealed-bit patterns are simply accounted for in the rank-(n−k) diagonal of *A*.

Notice that the properties of the equivocation matrix allow for the discovery of relationships between codes that hold for small codes and large codes as well. Studying patterns in small codes often yields great insight that can then be extended to codes of all sizes through analytical proofs. Additional insights into the existence of best codes that arise from the properties of equivocation matrices are given below in Section 5.

## 4. Searching for Best Codes

### 4.1. Notions of Equivalence in Wiretap Codes

In general coding theory, there is a notion of *code equivalence*. Consider the following definition.

**Definition** **3.** *Let $C_{\alpha }$ and $C_{\beta }$ be two (n,n−k) linear codes with generator matrices $G_{\alpha }$ and $G_{\beta }$, respectively. Then $C_{\alpha }$ and $C_{\beta }$ are equivalent if there exists an (n−k×n−k) invertible scrambling matrix F and (n×n) permutation matrix* Π *such that*

(19) $$G_{\beta }=FG_{\alpha }\Pi .$$

The codes $C_{\alpha }$ and $C_{\beta }$ have the same code parameters, e.g., code rate and minimum Hamming distance *d*. The effect of *F* is to perform elementary row operations on $G_{\alpha }$, which does not alter the set of codewords, but rather changes the mapping of messages to codewords when the codes are used for error correction [25,26]. When the codes are used for secrecy with the coset coding approach, the mapping of auxiliary messages to codewords is altered, but the sets of codewords in the cosets remain unchanged by *F*. The codewords of the two equivalent codes are identical up to a consistent reordering of the symbols in the codewords according to Π. This reordering holds for error correction and secrecy applications.

### 4.2. Search Space Reduction Techniques

In this section, we outline techniques for searching through all possible codes of small size. This requires a reduction in the search space of all codes so that (11) can be used to efficiently evaluate the average equivocation (2).

Let us consider the example code defined by the generator matrix in (5) with codewords arranged as in Table 1. Although this code is of a very small size, we can learn much from observing its structure and comparing it to other codes of the same size (i.e., n=4, and k=2). Suppose we take a naive approach to counting all linear (4,2) block codes and their accompanying coset structures. Using simple counting techniques we find that there are 105 different ways to choose 2 of 15 potential nonzero codewords. These choices form the bases of a code space, and hence define different generator matrices *G*. However, most of them are, in fact, isomorphisms of each other. Either they give the same code exactly, or some equivalent code.

**Lemma** **2.** *If generator matrices $G_{\alpha }$ and $G_{\beta }$ correspond to respective equivalent codes $C_{\alpha }$ and $C_{\beta }$, then the respective equivocation matrices formed by coset coding with these codes $A_{\alpha }$ and $A_{\beta }$ are identical.*

**Proof.**  The proof of this lemma is straightforward. From Definition 3, we see that $C_{\alpha }$ and $C_{\beta }$ are equivalent codes if $G_{\alpha }=FG_{\beta }\Pi$ for some invertible scrambling matrix *F* and a permutation matrix Π. As *F* does not change the cosets of the wiretap code, it can have no effect on the equivocation. For any revealed bit pattern $r=\{r_{1},r_{2},\ldots ,r_{\mu }\}$ with elements from 〚1,n〛, let π(r) be the set of indices resulting from the reordering of Π; that is, $\pi (r_{i})$ is the new index of the column ${\left(G_{\alpha }\right)}_{r_{i}}$ in $G_{\beta }$ for i∈〚1,μ〛. Thus, for any revealed bit pattern *r*, we have that

(20) $$\mathrm{rank}\left[{\left(G_{\beta }\right)}_{\pi (r)}\right]=\mathrm{rank}\left[{\left(G_{\alpha }\right)}_{r}\right]$$

since ${\left(G_{\beta }\right)}_{\pi (r)}$ and ${\left(FG_{\alpha }\right)}_{r}$ are equivalent up to a reordering of the columns. By noting that the equivocation matrix must consider (11) for all patterns *r*, the equivalence of $A_{\alpha }$ and $A_{\beta }$ is proved. □

Although the relationship in Lemma 2 is all that is needed to remove most (if not all) isomorphic generators from consideration when trying to analyze all codes of a specific size, we still benefit from known algorithms and/or shortcuts that can help us to remove a significant portion of isomorphic generators without having to test for equivalence. One way to remove a number of isomorphic codes from the list is to consider only systematic generators *G*. We recall that any generator matrix of a linear code can be put into systematic form through row operations and column pivots [25,26], and the resultant code is, therefore, equivalent to the original code. For the n=4 and k=2 case, this results in only 16 possible codes, and yet many of these are still isomorphic to each other leaving a list of codes with lingering redundancies.

Consider the graph-theoretic approach to algorithmically removing isomorphic codes by simply forming Tanner graphs, but based on generator matrices. For example, let us consider graphs corresponding to the following two systematic generators

(21) $$G_{A}=\left[\begin{array}{cccc} 1 & 0 & 1 & 1\\ 0 & 1 & 0 & 1 \end{array}\right],\;G_{B}=\left[\begin{array}{cccc} 1 & 0 & 1 & 0\\ 0 & 1 & 1 & 1 \end{array}\right].$$

**Figure 6:**
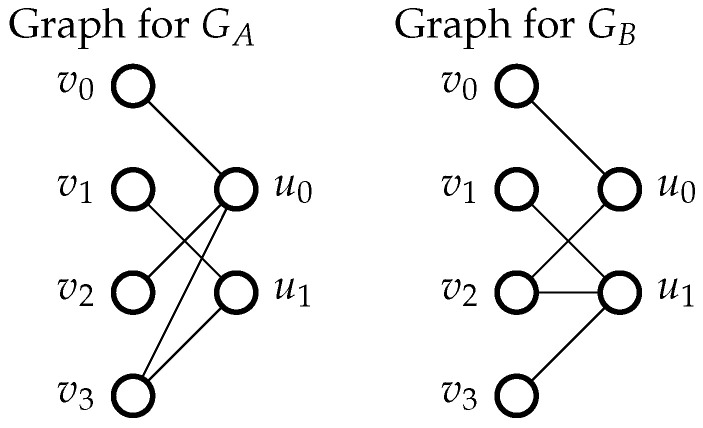
Two isomorphic bipartite graphs that represent equivalent generators.

Let $v_{0},v_{1},v_{2},v_{3}$ be column nodes and $u_{0},u_{1}$ be row nodes. Then connect node $u_{i}$ to node $v_{j}$ iff $G_{i,j}=1$. The generators then produce the graphs in Figure 6. The two graphs can be made equal by swapping labels in one of the graphs between $v_{0}$ & $v_{1}$, $v_{2}$ & $v_{3}$, and $u_{0}$ & $u_{1}$; therefore, they are isomorphic graphs, and the codes that represent them are likewise isomorphic codes. Removing all such graph isomorphisms results in a list of seven unique systematic generators of size 2×4. The respective systematic generator matrices for Codes 1 through 6 represent Codes 1 through 6 in Figure 2 and Figure 3. Codes 6 and 7 are given by the respective generator matrices

(22) $$G_{6}=\left[\begin{array}{cccc} 1 & 0 & 0 & 1\\ 0 & 1 & 1 & 1 \end{array}\right],\;G_{7}=\left[\begin{array}{cccc} 1 & 0 & 1 & 1\\ 0 & 1 & 1 & 1 \end{array}\right].$$

Although these codes do not create isomorphic graphs, they are clearly equivalent codes since $G_{6}$ can be created from $G_{7}$ with a single row operation followed by a column swap [25,26]. Furthermore, the code defined in (5) is also isomorphic to the codes produced by $G_{6}$ and $G_{7}$. The final redundant code can therefore be removed by inspection, leaving only the six unique codes. Examination of the equivocation matrices for all codes of this size shows that Code 6 is indeed best, in that it maximizes (15) for all μ∈〚0,n〛.

**Figure 7:**
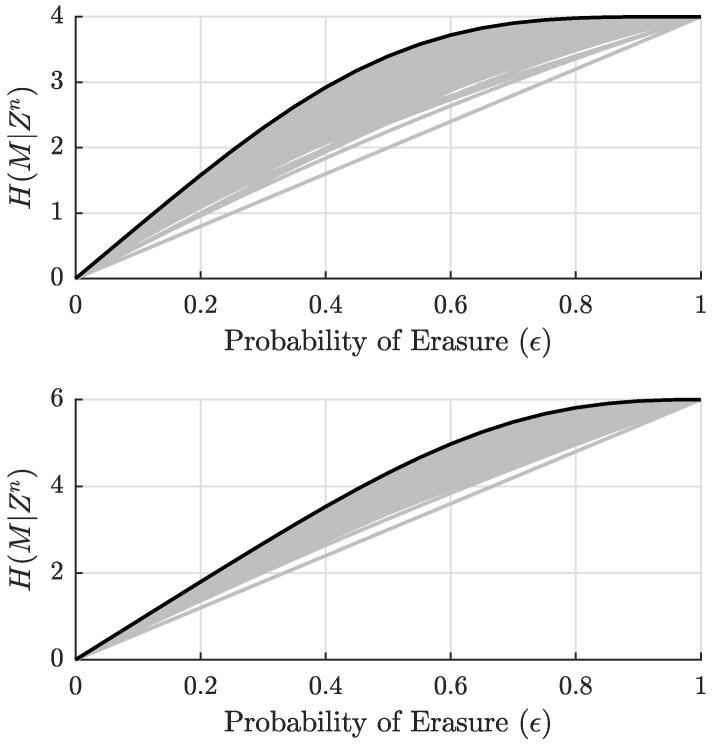
Equivocation of all n=8, k=4 secrecy codes (**top**) and all n=9, k=6 secrecy codes (**bottom**) as a function of Eve’s channel erasure probability ϵ. The best code exists and is given by the black line in each case.

Any set of algorithms that follow these guidelines for reducing the search space of all possible codes can be applied to any (n,k) design choices. Provided that both size parameters are small, numerical techniques can then create and compare all codes of the same size to identify the best ones. Although this approach will certainly not work for finding large best codes, it has been used effectively to identify several small best codes and various properties of best codes, which have then been proved analytically [32,33]. All (8,4) coset codes are given in the top plot of Figure 7, and all (9,6) coset codes are given in the bottom plot of Figure 7 by way of examples. Each of these cases has a best code that is provably-best through the sufficient condition of maximizing (15) for all μ∈〚0,μ〛. The best code for the (8,4) case has generator and equivocation matrices

(23) $$G=\left[\begin{array}{cccccccc} 1 & 0 & 0 & 0 & 0 & 1 & 1 & 1\\ 0 & 1 & 0 & 0 & 1 & 0 & 1 & 1\\ 0 & 0 & 1 & 0 & 1 & 1 & 0 & 1\\ 0 & 0 & 0 & 1 & 1 & 1 & 1 & 0 \end{array}\right],$$

(24) $$A=\left[\begin{array}{ccccccccc} 1 & 8 & 28 & 56 & 56 & 0 & 0 & 0 & 0\\ 0 & 0 & 0 & 0 & 14 & 56 & 0 & 0 & 0\\ 0 & 0 & 0 & 0 & 0 & 0 & 28 & 0 & 0\\ 0 & 0 & 0 & 0 & 0 & 0 & 0 & 8 & 0\\ 0 & 0 & 0 & 0 & 0 & 0 & 0 & 0 & 1 \end{array}\right],$$

while the best code for the (9,6) case has generator and equivocation matrices

(25) $$G=\left[\begin{array}{ccccccccc} 1 & 0 & 0 & 0 & 0 & 0 & 1 & 1 & 1\\ 0 & 1 & 0 & 0 & 1 & 1 & 0 & 1 & 1\\ 0 & 0 & 1 & 1 & 0 & 1 & 1 & 0 & 1 \end{array}\right],$$

(26) $$A=\left[\begin{array}{cccccccccc} 1 & 9 & 34 & 56 & 0 & 0 & 0 & 0 & 0 & 0\\ 0 & 0 & 2 & 28 & 117 & 0 & 0 & 0 & 0 & 0\\ 0 & 0 & 0 & 0 & 9 & 125 & 0 & 0 & 0 & 0\\ 0 & 0 & 0 & 0 & 0 & 1 & 84 & 0 & 0 & 0\\ 0 & 0 & 0 & 0 & 0 & 0 & 0 & 36 & 0 & 0\\ 0 & 0 & 0 & 0 & 0 & 0 & 0 & 0 & 9 & 0\\ 0 & 0 & 0 & 0 & 0 & 0 & 0 & 0 & 0 & 1 \end{array}\right].$$

## 5. Search Results: Best Codes Do Not Always Exist

It is natural to hypothesize that for every (n,k) pair for which a coset code exists, there should be a best code. The simulation results, however, have shown this idea to be false. In this section, we prove that not all (n,k) parameters for coset codes have a best code. We also present and prove a theorem based on the existence of best codes and equivocation matrices.

Consider coset codes for n=11 and k=6. The search through all codes of these size parameters is time consuming. *G* is 5×11, and the left-most 5×5 block in *G* can be fixed to $I_{5}$, the 5×5 identity, using the systematic technique from Section 4. However, there remain 30 bits in *G* to set, and we have no efficient technique to move quickly from unique code to unique code. With the search-space-reduction techniques from Section 4, we find only 20,755 codes that do not form graph isomorphisms, which can actually be evaluated relatively quickly once they are identified. When we evaluate all possible codes, we find two candidate codes to be best. Each maximizes (15) for a range of μ values, but no code maximizes (15) for all μ∈〚0,n〛. We call the two competing codes Code *L* and Code *R*. These codes are defined by their respective generator matrices

(27) $$G_{L}=\left[\begin{array}{ccccccccccc} 1 & 0 & 0 & 0 & 0 & 0 & 0 & 1 & 1 & 1 & 1\\ 0 & 1 & 0 & 0 & 0 & 1 & 1 & 0 & 0 & 1 & 1\\ 0 & 0 & 1 & 0 & 0 & 1 & 1 & 0 & 1 & 0 & 1\\ 0 & 0 & 0 & 1 & 0 & 0 & 1 & 1 & 1 & 0 & 1\\ 0 & 0 & 0 & 0 & 1 & 1 & 0 & 1 & 0 & 1 & 1 \end{array}\right],$$

and

(28) $$G_{R}=\left[\begin{array}{ccccccccccc} 1 & 0 & 0 & 0 & 0 & 0 & 0 & 1 & 1 & 1 & 1\\ 0 & 1 & 0 & 0 & 0 & 0 & 1 & 0 & 1 & 1 & 1\\ 0 & 0 & 1 & 0 & 0 & 1 & 1 & 1 & 0 & 1 & 1\\ 0 & 0 & 0 & 1 & 0 & 1 & 1 & 1 & 1 & 0 & 1\\ 0 & 0 & 0 & 0 & 1 & 1 & 1 & 1 & 1 & 1 & 0 \end{array}\right].$$

The equivocation matrices are

$$A_{L}=\left[\begin{array}{cccccccccccc} 1 & 11 & 55 & 165 & 305 & 287 & 0 & 0 & 0 & 0 & 0 & 0\\ 0 & 0 & 0 & 0 & 25 & 175 & 417 & 0 & 0 & 0 & 0 & 0\\ 0 & 0 & 0 & 0 & 0 & 0 & 45 & 325 & 0 & 0 & 0 & 0\\ 0 & 0 & 0 & 0 & 0 & 0 & 0 & 5 & 165 & 0 & 0 & 0\\ 0 & 0 & 0 & 0 & 0 & 0 & 0 & 0 & 0 & 55 & 0 & 0\\ 0 & 0 & 0 & 0 & 0 & 0 & 0 & 0 & 0 & 0 & 11 & 0\\ 0 & 0 & 0 & 0 & 0 & 0 & 0 & 0 & 0 & 0 & 0 & 1 \end{array}\right],$$

and

$$A_{R}=\left[\begin{array}{cccccccccccc} 1 & 11 & 55 & 163 & 300 & 288 & 0 & 0 & 0 & 0 & 0 & 0\\ 0 & 0 & 0 & 2 & 30 & 173 & 420 & 0 & 0 & 0 & 0 & 0\\ 0 & 0 & 0 & 0 & 0 & 1 & 42 & 326 & 0 & 0 & 0 & 0\\ 0 & 0 & 0 & 0 & 0 & 0 & 0 & 4 & 165 & 0 & 0 & 0\\ 0 & 0 & 0 & 0 & 0 & 0 & 0 & 0 & 0 & 55 & 0 & 0\\ 0 & 0 & 0 & 0 & 0 & 0 & 0 & 0 & 0 & 0 & 11 & 0\\ 0 & 0 & 0 & 0 & 0 & 0 & 0 & 0 & 0 & 0 & 0 & 1 \end{array}\right].$$

**Figure 8:**
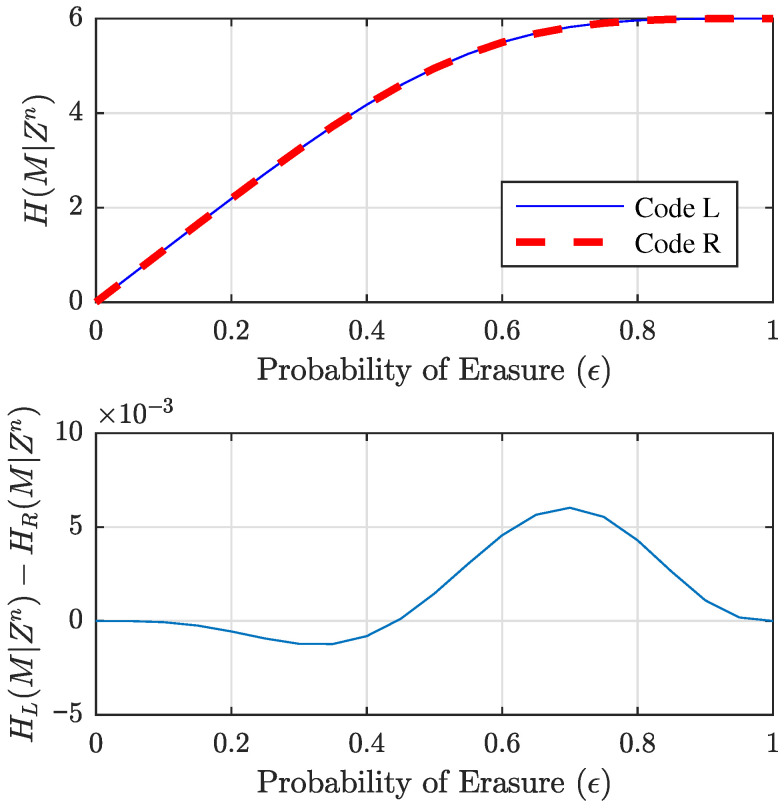
Equivocation curves for the two competing codes for the n=11, k=6 best code (**top**), and the difference between the two equivocation curves (**bottom**). Code *L* is best for the left-hand side of the equivocation matrix (right-hand side of the equivocation curve), and Code *R* is best for the right-hand side of the equivocation matrix (left-hand side of the equivocation curve).

Code *L* optimizes the *left* side of the equivocation matrix, while Code *R* optimizes the *right* side. Both manage to maximize (15) for μ=(n−k), but in different ways. Note that the number of full-rank patterns of size (n−k)=6 is 288 for Code *R* and only 287 for Code *L*. However, all remaining length-6 patterns have rank five for Code *L*, while exactly one pattern has rank four for Code *R*; thus, both codes have the same sum of ranks at μ=(n−k). Finally, we note that the actual equivocation curves for these codes cross. The curves are plotted in the top part of Figure 8, but the crossing point in ϵ can only be seen when the difference between the equivocation for Code *L* (given as $H_{L}\left(M|Z^{n}\right)$) and the equivocation for Code *R* (given as $H_{R}\left(M|Z^{n}\right)$) is plotted. This difference is given in the bottom plot of Figure 8, where we see Code *L* as superior (having higher equivocation) for higher ϵ. This high-ϵ region corresponds to the left side of the equivocation matrix, where a larger number of erasures are occurring. The crossing point occurs at roughly ϵ=0.444.

Now that we understand that there exist (n,k) size parameters that do not have a best code, we can state the following new theorem.

**Theorem** **1.** *Consider all possible coset codes for the size parameters (n,k). There exists a best coset code with these size parameters that satisfies the sufficient condition for best by maximizing (15) for all μ∈〚0,n〛 for use over the BEWC if and only if there exists a best code that satisfies the same sufficient condition for being classified as best for the size parameters (n,n−k).*

**Proof.**  The proof is straightforward using the dual relationship of the equivocation matrices given in Lemma 1. Recall that the lemma gives $A^{⊥}$ as being equal to *A* with the column order reversed while preserving the bottom left and upper right triangles of zeros. Since every code has a dual, the range of possible equivocation matrices for $A^{⊥}$ is the same as for *A* with this reordering. Optimality in one case implies optimality in the other case, and sub-optimality in one case implies sub-optimality in the other case. □

## 6. Principles of Code Design for Good Coset Codes of Any Size

In [33], we showed that Hamming codes and their duals (simplex codes) are almost surely best codes for their size parameters, and yet a complete proof of this almost sure fact is still missing, although we improve the result from [33] in Section 7. It would be good if a set of specific code families could be proved best, or even better if a generic algorithm for building best codes of any size parameters could be found. In this section, we present code design guidelines towards this goal. The end result is a set of design choices that can lead to very good codes of any size. We also present and prove a theorem that defines the relationship between maximum values of (15) and best codes in the reverse direction.

### 6.1. High-Level Design of Good/Best Codes

Let us consider again the implications of Lemma 1, and note that if an (n,k) coset code (built from an (n,n−k) linear block code C) has an associating equivocation matrix *A* that maximizes the sum in (15) for all μ∈〚0,n〛, then that code is provably-best for its size. Also, Theorem 1 states that the existence of the provably-best code C ensures the existence of a provably-best (n,n−k) coset code (built from an (n,k) linear block code $C^{⊥}$) such that the associating equivocation matrix $A^{⊥}$ likewise maximizes the sum in (15) for all μ∈〚0,n〛. Let *G* be a systematic generator matrix for C of the form

(29) $$G=\left[\begin{array}{cc} I_{n-k} & P \end{array}\right],$$

where *P* is an (n−k)×k submatrix of *G*. Then, it is well known [26] that an equivalent systematic generator for $C^{⊥}$ can be formed by

(30) $$G^{⊥}=\left[\begin{array}{cc} I_{k} & -P^{T} \end{array}\right].$$

A code design algorithm for C chooses columns of *P* or equivalently $-P^{T}$. Since we are only considering binary codes in this paper, we will drop the negative sign and consider only $P^{T}$.

**Figure 9:**
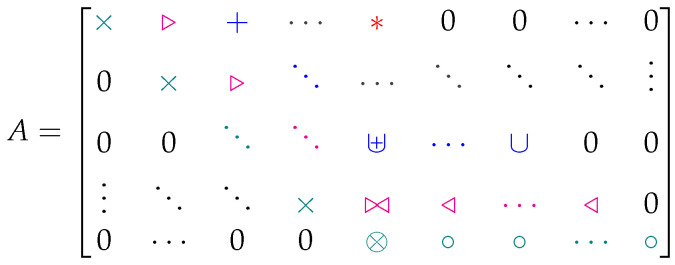
Pictorial representation of code design inspired by Lemma 1. Each diagonal can be designed by considering *G*, while each row can be designed by considering $G^{⊥}$. The designs meet in the middle at the μ=(n−k)th column. Symbols are merged in this column to indicate the meeting of designs.

Consider Figure 9 as we discuss design principles for good codes. Suppose columns of *P* are chosen to ensure no rank-zero patterns exist in the revealed-bit patterns for the coset code defined by C. This only amounts to not allowing any column of *P* to be all-zero, which sets an optimal structure for the elements in *A* along the diagonal dictated by the symbol ×, including the ⊗ entry. Since we know that optimal structures in $A^{⊥}$ also imply optimal structures in *A*, we can now shift our attention to $P^{T}$. Suppose we also ensure that no all-zero columns exist in $P^{T}$. This would fix the same optimal structure in $A^{⊥}$, which maps exactly to the entries of *A* marked by the ∘ symbol, including the ⊗ entry. Note that the ⊗ symbol is meant to be a merging of the × and ∘ symbols, since it is guaranteed optimal by both design steps. Thus, the first nonzero diagonal and the bottom row of *A* are optimized by ensuring no all-zero columns or rows are in *P*.

We can continue this design process to the next nonzero diagonal and row in *A* by ensuring no weight-one columns or rows are in *P* and that all rows and columns of *P* are unique. The problem is not necessarily the weight of the columns or rows, but rather that all weight-one columns already exist in *G* due to the systematic structure of the matrix. Thus, allowing any column of *P* to have weight one ensures a duplicate column in *G*, and the pattern associated with those two columns would have rank one in the submatrix of *G*, rather than rank two. If the size of *P* is such that it can be formed with all unique columns in *G* and $G^{⊥}$, we guarantee optimality in the entries of the equivocation matrix marked, respectively, by the symbols ▹ and ◃ along with the doubly designed entry marked by ⋈.

This process can continue, albeit at greater and greater complexity, by ensuring that the columns and rows of *P* do not introduce certain linear dependencies in the columns of *G* and $G^{⊥}$, respectively. At some point it will not be possible to ensure no dependencies of a particular size unless a maximum distance seperable (MDS) code exists for the size parameters in question [33], but effort towards removing many small linear dependencies in the columns of *G* and $G^{⊥}$ still results in better codes, and may lead to a best code in some cases.

Note also that this approach is similar to designing codes with good generalized Hamming weights outlined in [24]. The minimum distance of the dual code is the first generalized Hamming weight of the code itself, and the worst case equivocation (see works on the wiretap channel of type II [23]) is perfectly characterized by the generalized Hamming weights. When we couple these facts from [24] with Lemma 1, we find that codes which exhibit (along with their dual codes) large minimum distance and/or good distance properties will also provide good wiretap codes. We can use the same technique outlined above, wherein optimizing the distance properties of the dual code builds an optimum equivocation matrix from the left, and optimizing distance properties of the code itself builds an optimum equivocation matrix from the right.

### 6.2. Best Codes from the Outside in

In the previous subsection, we saw that an overall design idea for building good, and perhaps best, codes can operate from the outside entries of the equivocation matrix and work towards the inside entries. Here, we present a property of best codes that makes this idea rigorous using two theorems from [36]. First, let us rewrite the sum from (15) as

(31) $$s_{\mu }=\sum_{r\in R_{\mu }}\mathrm{rank}\left(G_{r}\right).$$

Then let $s=(s_{0},s_{1},\ldots ,s_{n})$ be the collection of these values for a code C with (n−k)×n generator matrix *G*. Let $s^{\prime }=\left(s_{0}^{\prime },s_{1}^{\prime },\ldots ,s_{n}^{\prime }\right)$ be the values from (31) calculated with the generator matrix for $C^{\prime }$ and let $s^{\prime \prime }=\left(s_{0}^{\prime \prime },s_{1}^{\prime \prime },\ldots ,s_{n}^{\prime \prime }\right)$ be the values from (31) calculated with the generator matrix for $C^{\prime \prime }$. Also, let γ be the smallest value of μ such that $s_{\mu }^{\prime }\neq s_{\mu }^{\prime \prime }$, and let δ be the largest value of μ such that $s_{\mu }^{\prime }\neq s_{\mu }^{\prime \prime }$. This implies that $s_{i}^{\prime }=s_{i}^{\prime \prime }$ for i∈{〚0,γ−1〛,〚δ+1,n〛}. Note that $s_{0}^{\prime }=s_{0}^{\prime \prime }=0$ and $s_{n}^{\prime }=s_{n}^{\prime \prime }=n-k$.

**Lemma** **3** (Corollary 1 from [36]). *If $s_{\gamma }^{\prime }>s_{\gamma }^{\prime \prime }$, then $C^{\prime \prime }$ cannot be the best code for its size. Similarly if $s_{\gamma }^{\prime }<s_{\gamma }^{\prime \prime }$, then $C^{\prime }$ cannot be the best code for its size.*

**Lemma** **4** (Corollary 2 from [36]). *If $s_{\delta }^{\prime }>s_{\gamma }^{\prime \prime }$, then $C^{\prime \prime }$ cannot be the best code for its size. Similarly if $s_{\delta }^{\prime }<s_{\delta }^{\prime \prime }$, then $C^{\prime }$ cannot be the best code for its size.*

From these two lemmas, our new theorem follows.

**Theorem** **2.** *If C is best for its size, then compared to any other code of the same size, say $C^{\prime \prime \prime }$ with $s^{\prime \prime \prime }=\left(s_{0}^{\prime \prime \prime },s_{1}^{\prime \prime \prime },\ldots ,s_{n}^{\prime \prime \prime }\right)$ being the values from (31) calculated with the generator matrix for $C^{\prime \prime \prime }$, it must be true that $s_{\gamma }>s_{\gamma }^{\prime \prime \prime }$ and that $s_{\delta }>s_{\delta }^{\prime \prime \prime }$.*

The proof is immediate from application of Lemmas 3 and 4. The theorem also completes our understanding, given the theoretical results to date, of the relationship between (15) and a best code. That is, any code that maximizes (15) for all μ∈〚0,n〛 is best; and any best code must at least maximize (15) for all $\mu \in \{〚0,\gamma^{*}-1〛,〚\delta^{*}+1,n〛\}$. For this statement, $\gamma^{*}$ is the maximum γ value obtained by letting $C^{\prime }$ be the best code and letting $C^{\prime \prime }$ range over all other codes of its size. Similarly, $\delta^{*}$ is the minimum δ value obtained by letting $C^{\prime }$ be the best code and letting $C^{\prime \prime }$ range over all other codes of its size.

## 7. On the Optimality of Simplex and Hamming Codes: Simplex Codes Are Better than Repeating Column Codes

In this section we prove that the simplex code maximizes (15) for all μ when compared to all codes of the same size with one column in *G* repeated any number of times.

**Theorem** **3.** *For any integer m≥2, $n=2^{m}-1$, and (n−k)=m, the $\left(2^{m}-1,m\right)$ simplex code is better for secrecy over the BEWC(ϵ) for all ϵ∈(0,1) than any code with exactly one column appearing more than once in the generator matrix.*

**Proof.**  The generator matrix *G* for the simplex code comprises all non-zero binary *m* tuples. Thus,

(32) $$G=\left[\begin{array}{cccc} g_{1} & g_{2} & ... & g_{n} \end{array}\right],$$

where $g_{i}\neq g_{j}$ for i≠j, and all columns have weight of at least 1. Suppose we also have a matrix $G^{\prime }$ such that

(33) $$G^{\prime }=\left[\begin{array}{cccc} g_{1}^{\prime } & g_{2}^{\prime } & ... & g_{n}^{\prime } \end{array}\right],$$

where

(34) $$g_{i}^{\prime }=\left\{\begin{array}{c} g_{i}\;\mathrm{for}\;3\leq i\leq n,\\ g_{2}\;\mathrm{for}\;i=1,2. \end{array}\right.$$

It was shown in [33] that the choice of index for the repeated column can be made without loss of generality. Similarly we have a matrix $G^{\prime \prime }$ defined as

(35) $$G^{\prime \prime }=\left[\begin{array}{cccc} g_{1}^{\prime \prime } & g_{2}^{\prime \prime } & ... & g_{n}^{\prime \prime } \end{array}\right],$$

where

(36) $$g_{i}^{\prime \prime }=\left\{\begin{array}{c} g_{i}\;\mathrm{for}\;4\leq i\leq n,\\ g_{2}\;\mathrm{for}\;i=1,2,3. \end{array}\right.$$

For j∈[[1,n]] and μ∈[[1,n]], we define a function ϕ as

(37) $$\phi \left(j,\mu \right)=\sum_{r\in \left[\;[1,n]\;\right]\setminus \left\{j\right\}:|r|=\mu -1}\mathrm{rank}\left[\begin{array}{cc} g_{j} & G_{r} \end{array}\right],$$

where $g_{j}$ is the *j*th column in *G*. As was shown in [33], all differences in rank between submatrices of *G* and $G^{\prime }$ are contained within the equations ϕ(1,μ) and $\phi^{\prime }\left(1,\mu \right)$ because all columns other than the first are identical. It was also shown in [33] that $\phi^{\prime }\left(1,\mu \right)<\phi \left(1,\mu \right)$.

If we consider $G^{\prime \prime }$, we see that similar to the previous case, there is only one difference in a column between $G^{\prime }$ and $G^{\prime \prime }$. As with the prior case, this implies that all differences between $G^{\prime }$ and $G^{\prime \prime }$ must be contained within a single phi function. In this case, all differences are contained within $\phi^{\prime \prime }\left(3,\mu \right)$. Due to the fact that within $G^{\prime \prime }$ columns 1, 2, and 3 are identical, it is clear that $\phi^{\prime \prime }\left(1,\mu \right)=\phi^{\prime \prime }\left(2,\mu \right)=\phi^{\prime \prime }\left(3,\mu \right)$.

Next, consider $\phi^{\prime }\left(1,\mu \right)$ and $\phi^{\prime \prime }\left(1,\mu \right)$. Notice that the only index that differs between $G^{\prime }$ and $G^{\prime \prime }$ is index 3 and, therefore,

(38) $$\phi_{G^{\prime }\setminus g_{3}^{\prime }}\left(1,\mu \right)=\phi_{G^{\prime \prime }\setminus g_{3}^{\prime \prime }}\left(1,\mu \right).$$

Because $g_{3}^{\prime \prime }=g_{2}^{\prime \prime }=g_{1}^{\prime \prime }$, adding $g_{3}^{\prime \prime }$ to $\phi_{G^{\prime \prime }\setminus g_{3}^{\prime \prime }}\left(1,\mu \right)$ cannot increase the rank of the subsets used to calculate $\phi^{\prime \prime }\left(1,\mu \right)$ and, therefore,

(39) $$\phi^{\prime \prime }\left(1,\mu \right)\leq \phi^{\prime }\left(1,\mu \right)<\phi \left(1,\mu \right).$$

This shows that $G^{\prime \prime }$ cannot have equivocation greater than $G^{\prime }$ and as such must be worse than *G*.

The inductive extension to any number of repeats of a single column is now straightforward. □

This result improves upon the best result to date in [33], which showed that simplex codes are better than codes with a single repeated column in *G*. Note that other codes with multiple unique columns appearing as repeats in *G* are unlikely to improve upon the equivocation over the simplex code. Additional repeats weaken the equivocation matrix in the μ=2 column, and these repeats are likely to harm other columns in *A* as well. Thus, we remain convinced that simplex codes are best for their size. We likewise continue to conjecture the optimality of Hamming codes due to their dual relation to simplex codes in Lemma 1.

## 8. Conclusions

This paper presents several results regarding the design of best coset codes for physical-layer security over the binary erasure wiretap channel. Equivocation matrices are defined and shown to be a useful tool in obtaining both practical and theoretical results regarding best codes. The search space reduction techniques showcased in this paper reduce the computation time required to find best codes of a given size. It was further shown that best codes do not exist for all size parameters of codes. The properties of best codes have also been extended to an *outside-in* property that shows that equivocation matrices of best codes must be optimal working from the outside columns of the matrix. Finally, we have shown that simplex codes are better than a family of codes with a single column repeated a number of times in the generator matrix.

This paper provides a framework on which future discoveries can be made. In particular, proving that simplex and Hamming codes are best for their size compared to all other codes may build on the results of this paper. Efficient algorithms for designing best codes may also be found by following the guidelines outlined in this work.

## Figures and Tables

**Table 1:** Code table for coset code defined by (5).

Coset		$m^{\prime }=\left[0\;0\right]$	$m^{\prime }=\left[0\;1\right]$	$m^{\prime }=\left[1\;0\right]$	$m^{\prime }=\left[1\;1\right]$
$C_{0}$	m=[00]	[0 0 0 0]	[0 0 1 1]	[1 1 0 1]	[1 1 1 0]
$C_{1}$	m=[01]	[1 0 1 0]	[1 0 0 1]	[0 1 1 1]	[0 1 0 0]
$C_{2}$	m=[10]	[1 1 0 0]	[1 1 1 1]	[0 0 0 1]	[0 0 1 0]
$C_{3}$	m=[11]	[0 1 1 0]	[0 1 0 1]	[1 0 1 1]	[1 0 0 0]

## Data Availability

The original contributions presented in this study are included in the article. Further inquiries can be directed to the corresponding author.
